# Identification of Novel Molecular Targets for Endometrial Cancer Using a Drill-Down LC-MS/MS Approach with iTRAQ

**DOI:** 10.1371/journal.pone.0016352

**Published:** 2011-01-31

**Authors:** Sébastien N. Voisin, Olga Krakovska, Ajay Matta, Leroi V. DeSouza, Alexander D. Romaschin, Terence J. Colgan, K. W. Michael Siu

**Affiliations:** 1 Department of Chemistry and Centre for Research in Mass Spectrometry, York University, Toronto, Ontario, Canada; 2 Division of Clinical Biochemistry, St. Michael's Hospital, Toronto, Ontario, Canada; 3 Department of Laboratory Medicine and Pathobiology, University of Toronto, Toronto, Ontario, Canada; 4 Pathology and Laboratory Medicine, Mount Sinai Hospital, Toronto, Ontario, Canada; The Research Institute for Children, United States of America

## Abstract

**Background:**

The number of patients with endometrial carcinoma (EmCa) with advanced stage or high histological grade is increasing and prognosis has not improved for over the last decade. There is an urgent need for the discovery of novel molecular targets for diagnosis, prognosis and treatment of EmCa, which will have the potential to improve the clinical strategy and outcome of this disease.

**Methodology and Results:**

We used a “drill-down” proteomics approach to facilitate the identification of novel molecular targets for diagnosis, prognosis and/or therapeutic intervention for EmCa. Based on peptide ions identified and their retention times in the first LC-MS/MS analysis, an exclusion list was generated for subsequent iterations. A total of 1529 proteins have been identified below the Proteinpilot® 5% error threshold from the seven sets of iTRAQ experiments performed. On average, the second iteration added 78% new peptides to those identified after the first run, while the third iteration added 36% additional peptides. Of the 1529 proteins identified, only 40 satisfied our criteria for significant differential expression in EmCa in comparison to normal proliferative tissues. These proteins included metabolic enzymes (pyruvate kinase M2 and lactate dehydrogenase A); calcium binding proteins (S100A6, calcyphosine and calumenin), and proteins involved in regulating inflammation, proliferation and invasion (annexin A1, interleukin enhancer-binding factor 3, alpha-1-antitrypsin, macrophage capping protein and cathepsin B). Network analyses revealed regulation of these molecular targets by c-myc, Her2/neu and TNF alpha, suggesting intervention with these pathways may be a promising strategy for the development of novel molecular targeted therapies for EmCa.

**Conclusions:**

Our analyses revealed the significance of drill-down proteomics approach in combination with iTRAQ to overcome some of the limitations of current proteomics strategies. This study led to the identification of a number of novel molecular targets having therapeutic potential for targeted molecular therapies for endometrial carcinoma.

## Introduction

Endometrial carcinoma (EmCa) is the fourth most-prevalent cancer in women in North America, with 42160 new cases and 7780 deaths expected this year in the U.S. alone [1]. There are two major types of endometrial cancer: Type I EmCa of endometrioid histology and Type II EmCa which is serous or clear cell in morphology, the latter type typically being the more aggressive of the two. Type I EmCa is the common form of endometrial cancer, constituting about 70–80% of the total cases [2], [3]. Diagnosis is mostly based on histological examination of tissues obtained after a biopsy, an invasive procedure typically performed as a result of investigative diagnosis upon abnormal uterine bleeding at presentation. Endometrial carcinomas have been primarily treated by means of surgery with additional radiation and/or chemotherapy, and relatively favorable outcomes have been attained provided the cancers are detected early. However, the number of patients with EmCa in an advanced stage or high histological grade, which is indicative of a poor prognosis, is increasing [2], [4]. There is an urgent need for the discovery of novel molecular targets for the diagnosis, prognosis and treatment of EmCa, which will have the potential to improve the clinical strategy and outcome of this disease.

Recently, there has been intense interest in the study of global protein expression, and proteomic approaches appear to present a new strategy for cancer research and the identification of new biomarkers for clinical application. Numerous proteomic studies undertaken in recent years have attempted to discover potential cancer biomarkers through differential protein screening using cancer and non-cancerous tissues [5]–[9]. One of the most widely used technologies for biomarker discovery is a bottom-up approach that involves chemical labeling of peptides resulting from enzymatic digestion of sample proteins, followed by mixing of the control and test samples prior to liquid chromatography-tandem mass spectrometry (LC-MS/MS) analysis to ensure identical treatment of the samples [6], [7], [9]. Among the more common of these labeling reagents in use currently are the isobaric tags for relative and absolute quantification (iTRAQ) [10]. Tagging enables distinguishing of otherwise identical peptides originating from individual samples in the mixture via reporter ions formed from these tags, which permit relative quantification of the given peptide in the samples, and thereby the corresponding protein. Due to the complexity of tissue samples, a pre-fractionation of the samples using strong cation exchange (SCX) is typically performed, prior to analytical separation on a nanoscale reverse-phase (RP) column, which is coupled online with the mass spectrometer. Despite this two-dimensional chromatographic separation, there is still a tendency for a large number of peptides to co-elute during the RP separation because of sample complexity. As a result, the mass spectrometer is typically only able to examine a fraction of the peptides due to time constraints. In an automated data acquisition, the MS software tends to favor analysis of the most abundant peptides at the expense of co-eluting peptides of lower abundances. As many cancer-relevant proteins, including signaling and regulatory proteins, are typically expressed in low concentrations, this bottom-up, shotgun approach tends to miss out on acquiring the most-valuable information [11]. A possible solution to this issue is an iterative analysis of the same sample, coupled with an exclusion list of identified peptides that is generated after each run and used to inform the choice of ions that are targeted for MS/MS analysis during subsequent runs. This strategy forces the mass spectrometer to target new, less-abundant peptides for MS/MS analysis during each successive iteration. Variations of this approach have been reported for matrix-assisted laser desorption/ionization (MALDI) MS/MS and ESI-MS/MS [12], [13].

In the study, we used an iterative analysis, referred herein as the “drill down approach”, to facilitate the identification of novel molecular targets for diagnosis, prognosis and/or therapeutic intervention for endometrial cancer. The major objective of this drill-down method is to unearth more peptides. After the first LC-MS/MS analysis, the identified peptide ions and their retention time were added to generate an exclusion list for further iterations in LC-MS/MS analysis. Our approach is conceptually similar to the “selective precursor ion exclusion” (sel-PIE) method described earlier by Wang *et al.* [11]. To our knowledge, this is the first time that such a combination of protein quantification using iTRAQ labeling and iterative analysis approach has been used for discovery of cancer biomarkers.

## Results

### Identification of proteins using iterative analysis with iTRAQ

**Figure 1:**
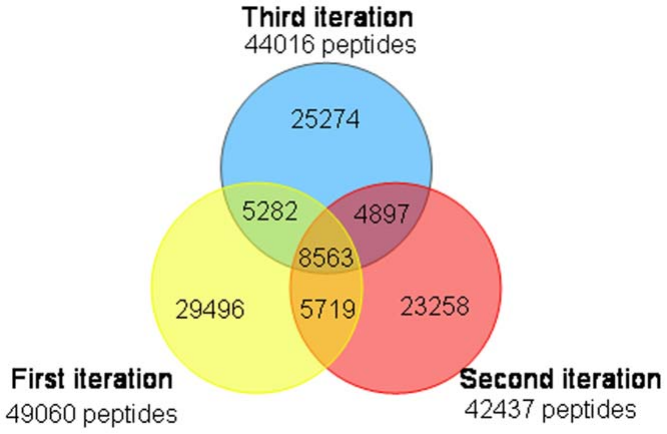
Number of unique peptides identified in one or more iterations, sum of all sets. On average, the second iteration added 78% new peptides to those identified after the first run, while the third iteration added 36% more peptides. Within a given set, about 12% of the peptides were common to any two successive iterations; however, only 3 to 6% of the peptides were identified in all three iterations.

**Figure 2:**
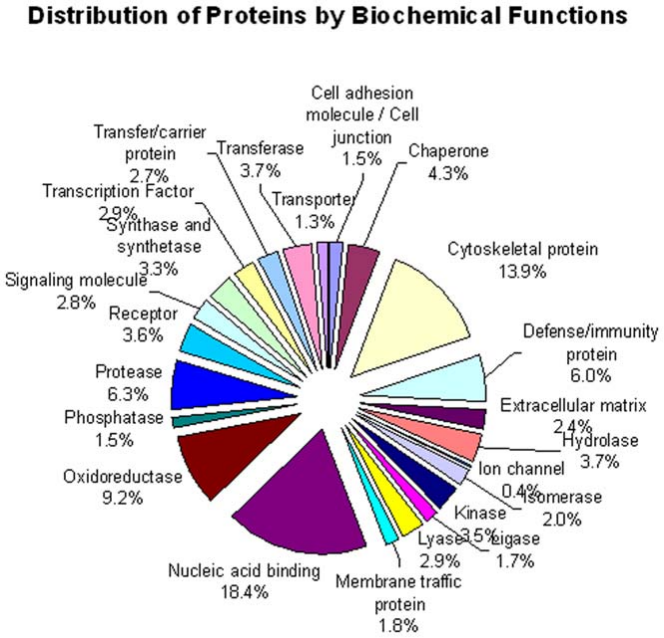
Distribution of proteins according to their cellular functions.

A total of 1529 proteins have been identified by Proteinpilot® at a confidence level of 95% from the seven sets of iTRAQ experiments carried out in this study. The first run of all the fractions identified a total of 1137 non-redundant proteins, with the second and third iterations contributing the remaining 392 non-redundant proteins. On average, the second iteration added 78% new peptides to those identified after the first run, while the third iteration added 36% more peptides as shown in Figure 1 and Table 1. Within a given set, about 12% of the peptides were common to any two successive iterations; however, only 3 to 6% of the peptides were identified in all three iterations. As expected, these additional peptides improved the coverage of identified proteins. Indeed, the second iteration added 34% new proteins on average, while the third iteration added only 14% to the combined list from iterations 1 and 2; thus there was little incentive to perform further iterations. 40% of the proteins identified during the first run were also identified in the next two iterations (Table 1). Of the 1529 proteins, 623 were identified by a single peptide; 423 of which showed ≥99% in confidence. The remaining 906 proteins were identified on average with six peptides per protein, considering only peptides with ≥95% confidence scores. Of the 1529 proteins identified in this study, 1260 proteins (i.e. 82%) had iTRAQ ratios reported for EmCa tissue samples. A full list of the identified proteins and their mean iTRAQ ratios is available in Table S1. A pie chart showing the distribution in cellular functions of these proteins is shown in Figure 2.

**Table 1:** Comparison of identified protein numbers across iterative analyses of iTRAQ sets^a^.

	Total proteins		Additional proteins			Additional		Common
	All runs	Iter 1	Iter 2	Iter 3	Common to all iterations	Iter 2/1	Iter 3/1+2	Common to all iterations
Set 1	888	787	40	61	122	5.1%	7.4%	15.5%
Set 2	635	434	122	79	162	28.1%	14.2%	37.3%
Set 3	520	408	14	98	136	3.4%	23.2%	33.3%
Set 4	207	148	51	8	48	34.5%	4.0%	32.4%
Set 5	263	161	49	53	91	30.4%	25.2%	56.5%
Set 6	558	227	265	66	141	116.7%	13.4%	62.1%
Set 7	554	412	81	61	190	19.7%	12.4%	46.1%
					Average:	34.0%	14.3%	40.5%

a Numbers correspond to the number of unique proteins identified; percentages refer to new proteins over existing ones.

### Identification of differentially expressed proteins in EmCa

To determine differentially expressed proteins that may serve as potential molecular targets for the evaluation of diagnosis and/or prognosis of EmCa, all proteins (n = 1529) identified in this study were evaluated using the criteria described in the Material & Methods section. A list of 40 proteins that may serve as potential biomarkers for EmCa is shown in Table 2. Of these 40 biomarker candidates, 38 were identified with a minimum of two peptides. The two exceptions (S100 calcium-binding protein A6 and beta-2-glycoprotein 1) were included as they were identified by a peptide with ≥99% in confidence, and manual inspection showed excellent MS/MS spectral quality (Figures S1 and S2). Individual iTRAQ values for protein quantification are listed in Table S2a; a list of all identified peptides with a Proteinpilot confidence level ≥95% is available in Table S3. Table S4 lists the iterations in the sample analysis where proteins in Table 2 were quantified. As the EmCa and normal proliferative tissues were obtained from different individuals and were, therefore, not identical (i.e., the analyses were not replicates), the typical concept of standard deviation in quantitative analysis may be misleading as a measure of analytical quality. We have, therefore, opted to express the distributions of combined analytical and individual variability in the following way: in our analyses, 28% of individual iTRAQ values deviate within ±10% from their means, 54% within ±20%, and 88% within ±50% (see Table S2b for details). These distributions are in support of our hypothesis that a 50% change in iTRAQ ratios is indicative of differential expression. Indeed, we identified a group of 16 additional proteins which just missed the cutoff of our criteria for differential expression and which may yet qualify after inclusion of additional samples (Table 3).

**Table 2:** Mean iTRAQ ratios for potential EmCa molecular targets.

Accession Number	Protein Name	EmCa tissues	Normal tissues	p-value	PPV	AUC
P62851	40S ribosomal protein S25	1.91	1.06	0.01	0.71	0.74
P07108	Diazepam binding inhibitor	1.60	0.97	0.002	0.88	0.81
P23526	Adenosylhomocysteinase	1.62	0.99	0.002	0.80	0.74
P01009	Alpha-1 antitrypsin	0.56	0.99	0.00	0.89	0.85
P02765	Alpha-2-HS-glycoprotein	0.56	0.99	0.01	0.71	0.77
P01023	Alpha-2-macroglobulin	0.59	0.92	0.002	0.75	0.79
O95994	Anterior gradient protein 2 homolog	1.82	1.04	0.025	0.71	0.60
P02647	Apolipoprotein A-I	0.67	0.99	0.025	0.83	0.88
P02652	Apolipoprotein A-II	0.55	0.92	0.025	0.71	0.74
Q15121	Astrocytic phosphoprotein PEA-15	1.98	0.76	0.01	0.80	0.73
P02749	Beta-2-glycoprotein 1	0.50	1.01	0.005	0.67	0.69
Q13938	Calcyphosine	1.60	0.95	0.00	0.89	0.85
O43852	Calumenin (Crocalbin)	0.45	0.93	0.002	1.00	1.00
P07858	Cathepsin B	1.61	1.05	0.002	0.88	0.84
P00450	Ceruloplasmin	0.62	1.12	0.002	0.83	0.86
Q99832	Chaperonin containing TCP1, subunit 7 (Eta)	1.59	0.95	0.025	1.00	0.67
P02452	Collagen, type I, alpha 1	0.52	0.99	0.002	0.73	0.75
P08123	Collagen, type I, alpha 2	0.56	1.02	0.002	0.73	0.81
Q16555	Dihydropyrimidinase-like 2	0.64	1.01	0.00	0.80	0.80
P05198	Eukaryotic translation initiation factor 2 subunit 1	1.90	1.03	0.05	0.83	0.77
P47756	F-actin capping protein subunit beta (CapZ beta)	1.57	0.88	0.005	0.75	0.63
P02671	Fibrinogen alpha chain	0.54	0.89	0.005	0.62	0.65
P02675	Fibrinogen beta chain	0.50	0.93	0.005	0.64	0.71
P02679	Fibrinogen gamma chain	0.36	0.75	0.025	0.75	0.77
P23142	Fibulin-1	0.47	0.97	0.01	0.80	0.73
Q14315	Filamin-C	0.53	0.95	0.025	0.67	0.63
P00738	Haptoglobin	0.58	0.87	0.025	0.75	0.79
P69905	Hemoglobin subunit alpha	0.55	0.93	0.005	0.58	0.60
P19823	Inter-alpha (Globulin) inhibitor, H2 polypeptide	0.56	0.92	0.025	0.67	0.76
Q12906	Interleukin enhancer-binding factor 3	1.79	1.18	0.05	0.75	0.69
P05787	Keratin, type II cytoskeletal 8	1.98	1.01	0.00	1.00	1.00
P40121	Macrophage capping protein (CAP-G)	1.67	0.91	0.00	1.00	1.00
P29966	Myristoylated alanine-rich C-kinase substrate (MARCKS)	0.65	1.08	0.00	1.00	0.87
O94788	Retinal dehydrogenase 2	0.41	0.91	0.00	0.80	0.87
P06703	S100 calcium-binding protein A6 (Calcyclin)	1.56	0.93	0.005	0.83	0.70
Q6FHJ7	Secreted frizzled-related protein 4	0.43	0.72	0.01	0.67	0.77
P50454	Serpin H1 (47 kDa heat shock protein)	0.34	0.96	0.005	0.78	0.80
P02768	Serum albumin	0.54	0.97	0.001	0.80	0.80
P02787	Transferrin	0.56	0.99	0.00	0.80	0.80
P02774	Vitamin D-binding protein	0.58	1.02	0.00	0.86	0.89

**Table 3:** Additional potential EmCa biomarker candidates^a^.

Accession Number	Protein Name	EmCa No. of samples^b^	EmCa mean iTRAQ ratio	Normal No. of samples^b^	Normal mean iTRAQ ratio
Q01518	Adenylyl cyclase-associated protein 1 (CAP 1)	5	1.43	7	1.05
P04217	Alpha-1-B glycoprotein	6	0.69	6	1.07
P04083	Annexin A1	10	1.49	10	1.07
O00299	Chloride intracellular channel protein 1	5	1.47	7	1.05
P0C0L4	Complement component 4A	7	0.68	8	1.03
P12277	Creatine kinase B-type	10	0.69	10	1.00
P21333	Filamin A, alpha	10	0.67	10	0.90
P04792	Heat-shock protein beta-1 (HSP 27)	10	0.68	10	0.96
P02790	Hemopexin (Beta-1B-glycoprotein)	5	0.70	7	1.04
P09651	Heterogeneous nuclear ribonucleoprotein A1	8	1.42	9	0.85
P05783	Keratin, type I cytoskeletal 18	9	1.43	6	0.87
P00338	L-lactate dehydrogenase A chain	10	1.45	10	1.01
P19338	Nucleolin (Protein C23)	7	1.42	5	0.85
P14618	Pyruvate kinase isozymes M1/M2	10	1.43	10	1.00
Q8NBS9	Thioredoxin domain-containing protein 5	9	0.70	9	0.99
Q6NUR7	Villin 2	7	1.47	8	1.08

a Differentially expressed proteins that satisfy all but one criteria described in Material & Method; these proteins all have changes in expression between 40 and 50%, or expression ratios between 1.4 and 1.5 or between 0.67 and 0.71.

b Number of samples observed, out of 10.

### Evaluation of diagnostic potential of differentially expressed proteins in EmCa

Other parameters relevant to diagnostic potentials were also evaluated. These include positive predictive values (PPVs) and areas under the curve (AUCs) available from receiver operating characteristics (ROC) analysis. Values for the individual biomarker candidates are listed in Table 2.

### Verification of differential expression of proteins in EmCa tissues

**Figure 3:**
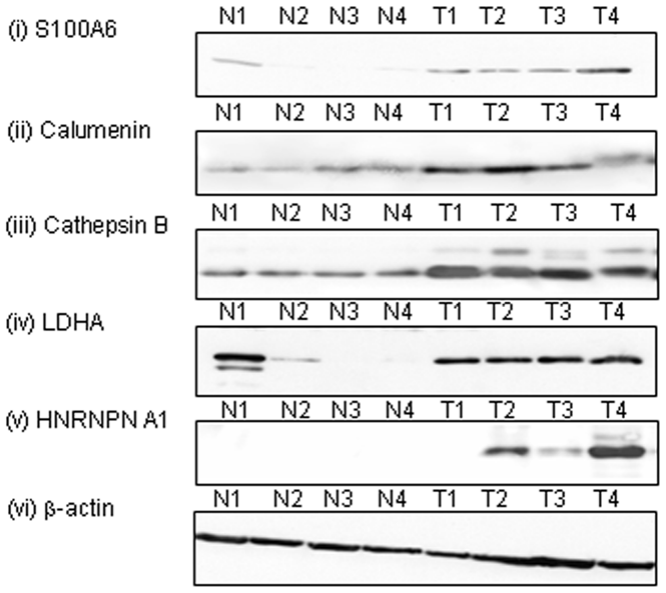
Verification of over-expression of proteins in EmCa tissues by Western blot analysis. Equal amounts of proteins (50 µg/lane) were resolved on 10% sodium dodecyl sulfate (SDS)-polyacrylamide gel and then electro-transferred onto polyvinylidene-difluoride (PVDF) membrane. After blocking, blots were incubated with respective mouse monoclonal antibody at appropriate dilutions at 4°C O/N. Membranes were incubated for 2 h at room temperature with secondary antibody. Protein bands were detected by the enhanced chemiluminescence method (GE Health Care) on Kodak hyperfilm. Western blot analysis showed over expression of (i) S100A6, (ii) calumenin, (iii) cathepsin B, (iv) lactate dehydrogenase A (LDHA), and (v) heterogeneous protein A1 (HNRNPA1) in endometrial cancer tissues (T1, T2, T3 and T4) in comparison to normal endometrium (N1, N2, N3 and N4). β-actin served as a loading control showing equal protein amounts in each lane.

**Figure 4:**
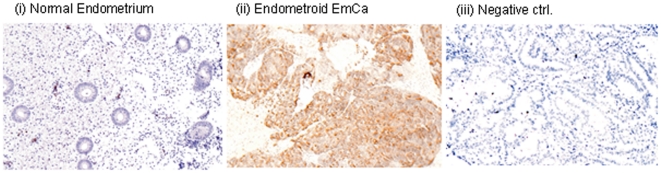
Immunohistochemistry of S100A6 protein in EmCa tissues and normal endometrium. Immunohistochemical analysis of S100A6 protein was carried out in independent paraffin-embedded tissue sections of endometrial cancer and normal endometrium (n = 5 each) as described in Materials and Methods. Panel shows (a) no immunostaining of S100A6 protein in normal endometrium; (b) endometrial cancer showing cytoplasmic expression of S100A6 protein; and (c) negative control showing no expression of S100A6 protein.

The overexpression of selected biomarker candidates: cathepsin B, calumenin, S100A6, lactate dehydrogenase A (LDHA), and HNRNPA1 in EmCa tissues were verified by Western blot analyses in a subset of the same tissue samples used for iTRAQ LC-MS/MS. Figure 3 shows a comparison of four EmCa tissues (T) with four normal endometria (N) for the five biomarker candidates with β-actin serving as a loading control. Furthermore, immunohistochemical analyses in an independent set of tissue samples (n = 5 each) revealed intense cytoplasmic and/or nuclear immunostaining of S100A6 protein in tumor cells of endometrioid EmCa tissue sections (typical results are shown in Figure 4), whereas no significant immunostaining was observed in the epithelial cells of normal proliferative endometria.

### Network Analysis

**Figure 5:**
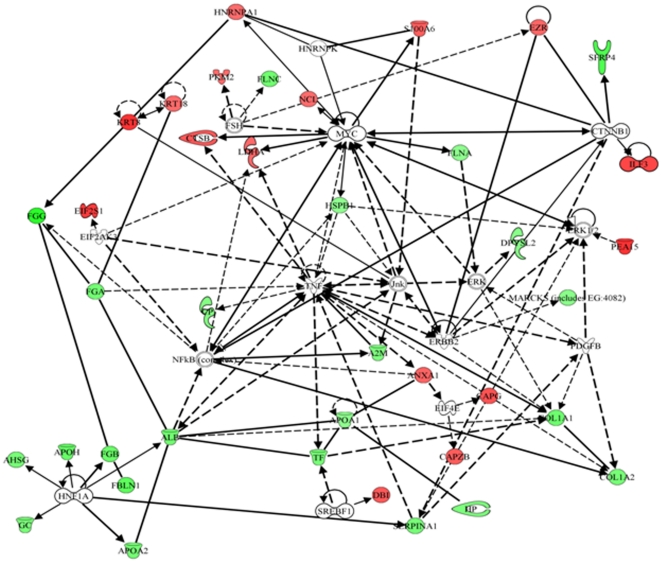
Network analysis. The potential novel molecular targets identified in this study were subjected to pathway analyses using Ingenuity Pathways Analysis (IPA) software, Version 7.5. The figure shows direct (bold lines) and indirect (dotted lines) regulations (→)/interactions (--) among the biomarkers identified in this study and other significantly associated proteins. These networks reveal involvement of key proteins including TNFα, NFκB, c-myc, Her2/Neu, β-catenin, Jnk and Erk1/2 proteins that regulate inflammation, survival and proliferation.

We performed Ingenuity Pathway Analysis (IPA) to generate networks showing direct and indirect regulations/interactions among proteins identified in this study. These networks show involvement of key players including TNF alpha, NFκB, c-myc, Her2/Neu, β-catenin, and Erk1/2 proteins, which regulate inflammation, and the survival and proliferation of tumor cells (Figure 5). Most of the molecular targets identified in this study are regulated by c-myc, Her2/neu, and TNF α, thus suggesting intervention of these pathways may provide a means to the development of molecular targeted therapies for endometrial cancer.

## Discussion

There has been considerable recent interest in the identification of potential cancer markers for diagnosis and prognosis via differential proteomic analysis. The various issues, pitfalls, and successes of these proteomics-based biomarker studies have been discussed and reviewed [14]–[17]. Our study led to the identification of 40 proteins showing significant differential expression in EmCa in comparison with normal proliferative tissues. These proteins include metabolic enzymes [pyruvate kinase M2 (PKM2) and lactate dehydrogenase A (LDHA)]; calcium binding proteins (S100A6, calcyphosine and calumenin); and proteins involved in regulating inflammation, proliferation and invasion [annexin A1 (ANXA1), interleukin enhancer-binding factor 3 (ILF3), alpha-1-antitrypsin (AAT), macrophage capping protein (CAPG) and cathepsin B]. Several reports have shown the influence of estrogen receptor (ER) and p53 on expression of these proteins, which warrants verification of these observations in EmCa tissues on a larger scale. Among the proteins identified in this study, we have previously reported altered expression of AAT, CAPG, pyruvate kinase M1/M2, and creatinase kinase B. Two of these proteins (pyruvate kinase M2 and creatinase kinase B) have been included here for consideration, as additional manual investigation of the data revealed that while the expression ratio changes for these two proteins were just under 50% (pyruvate kinase M1/M2, 1.43; and creatinase kinase B, 0.69), they did meet the other two criteria (see Materials and Methods). Furthermore, an independent study using immunohistochemistry on a tissue microarray (n = 148 patients) carried by our group demonstrated the remarkable potential of AAT and PKM2 as diagnostic biomarkers for EmCa [18]–[20]. It is noteworthy that increased amount of tumor PKM2 has also been reported in the tumor cells and EDTA plasma of patients with cancers of the kidney, lung, breast, cervical and gastrointestinal tract, as well as in stool samples of patients with colorectal and stomach cancer [21]. Thus PKM2 may act as a general indicator of malignancy, rather than being specific to EmCa. This association with tumorigenesis in general is, perhaps, understandable in light of PKM2's function [21]. The switch to the M2 isoform of pyruvate kinase in tumor cells is necessary to induce the Warburg effect. Increased expression of PKM2 contributes to a metabolic environment that is amenable to cell proliferation under hypoxic conditions and promotes tumor cell growth [22], [23]. Thus, PKM2 acts as a metabolic sensor, which allows the tumor cell to adapt its metabolism to variations in the supply of nutrients. Interestingly, LDHA also showed overexpression in EmCa tissues in this study, and PKM2 is known to preferentially shuttles pyruvate to lactate dehydrogenase [24]. Tyrosine phosphorylation of lactate dehydrogenase facilitates protein binding to PKM2, thereby channeling the product of pyruvate kinase to lactate [25]. This helps in generating nicotinamide adenine dinucleotide (NAD^+^) required for maintaining high glycolytic flux in tumor cells. This metabolic conversion makes glycolysis self-sufficient, as long as elevated glucose uptake is feasible. A high glycolytic rate provides synthetic intermediates to rapidly proliferating tumor cells, required to replicate cell biomass and genome at each cell division [24], [26].

Another important group of proteins found to be deregulated in EmCa tissues includes four calcium-binding proteins: S100A6, calcyphosine (CAPS), calumenin (CALU), and annexin A1 (Table 2). S100A6 is predominantly a cytoplasmic protein, but in the presence of Ca^2+^, it can associate with the plasma membrane and the nuclear envelope. Our immunohistochemical analysis showed cytoplasmic and/or nuclear staining of S100A6, predominantly in tumor cells of endometrioid EmCa. This is further supported by the presence of S100A6 protein in the cytoplasm and nuclei of lung, skin, and pancreatic ductal adenocarcinoma cells [27]–[29]. S100A6 plays an important role in cytoskeletal reorganization, invasion, survival and proliferation by regulating functions of several molecular targets, including p53, β-catenin, annexins, tropomyosin, calponin, calcyclin-binding protein/Siah-1-interacting protein (CacyBP/SIP), and Hsp90/Hsp70-organizing protein (Hop) [30]–[33]. Overexpression of S100A6 has been associated with poor prognosis in lung, gastric and pancreatic cancer [27], [31], [33]. Of the four Ca^2+^ regulatory proteins found to be differentially expressed in this study, only calcyphosine has previously been reported to be associated with poor prognosis in EmCa patients [34]. Annexin A1 (ANXA1), found to be overexpressed in EmCa tissues in this study, is an endogenous anti-inflammatory protein. Annexins are a family of Ca^2+^/lipid-binding proteins involved in diverse cellular functions, such as membrane aggregation, inflammation, phagocytosis, proliferation, and apoptosis [35]. Together, these results suggest that the calcium-phosphatidylinositol and cyclic AMP cascades may play an important role in the regulation of cell function, proliferation, and differentiation in endometrial carcinogenesis.

Notably, our study also indicates an important role of inflammation regulatory and RNA binding proteins in high-grade EmCa. Upregulation of the aforementioned annexin A1 together with downregulation of apolipoproteins, fibrinogens and haptoglobin suggests suppression of the inflammatory process in tissues surrounding the tumor. Interleukin enhancer-binding factor 3 (ILF3 or NF90) and heterogeneous ribonucleoprotein A1 (hnRNP A1) are RNA binding proteins that regulate expression of several proteins involved in survival and proliferation of tumor cells [36]–[38]. Among others, overexpression of serpin H1, F-actin capping protein subunit beta (CAPZB), macrophage capping protein (CAPG), villin 2 (EZR), and cathepsin B (CTSB) are known to promote cell motility and invasion in tumor tissues [39]–[42]. Among the potential molecular markers that could aid in diagnosis or prognosis of EmCa, some such as PKM2, LDHA and cathepsin B have already been explored for their therapeutic potential in other cancers. Inhibition of PKM2 and LDHA, using short interfering RNA (siRNA) or inhibitors, showed reduced cell proliferation due to induction of oxidative stress resulting in apoptosis [43]–[47]. Furthermore, siRNA-mediated downregulation of PKM2 sensitized lung cancer cells to cisplatin and doxorubicin. The potential of these proteins to serve as targets for novel molecular target-based therapies, therefore, needs to be determined in the context of endometrial cancer as well.

In this study, the drill-down approach improved the number of proteins identified, although a core set of peptides was detected in all runs of any given sample. These instances of repeated detection are attributable to shifts in retention time, peak tailing, multiple charges, and modifications (e.g., de-amidation and methionine oxidation). Following the analysis of the first iTRAQ set, we improved the precision of fraction injection and widened the exclusion windows for both time and *m/z*, from ±5 to ±7 min, and from 100 mDa to 120 ppm, respectively to mitigate some of these challenges. We chose not to exclude ions based on differences of charge or modification, as this would have increased the exclusion list beyond a practical size, and additionally we reasoned that some redundancy based on differences of charge or modification may serve to increase the confidence of identification. Thus the iterative analyses we employed struck a balance between the depth of analysis and tractability. However, this identification via multiple peptides meant that the number of identified proteins probably increased at a slower rate than would have been possible had we implemented the exclusion of different charge states and modifications. Interestingly, the total number of proteins identified in this study were comparable to those identified in our previous study (1529 versus 1388 proteins, respectively), despite working here with only half of the amount of starting material (100 versus 200 µg/sample used earlier) [18].

In conclusion, our analysis clearly reveals the significance of drill-down proteomic approach in combination with iTRAQ in identifying biomarker candidates for endometrial cancer. This study successfully reveals novel differentially expressed proteins that could serve as molecular targets in diagnosis and/or prognosis of endometrioid EmCa tissues. Some of these proteins exhibit potentials as molecular targets for therapeutics.

## Materials and Methods

### Ethics Statement

Endometrial tissues were retrieved from an in-house, dedicated research endometrial-tissue bank as described earlier [18]. The collection and use of these materials were approved by the Research Ethics Boards of York University, Mount Sinai Hospital, University Health Network, and North York General Hospital. The samples originated from patients undergoing hysterectomies or other clinical procedures involving biopsies. All these samples were obtained after written informed consent by all participants involved in this study.

### Samples and Reagents

Unless stated otherwise, all reagents were available from Sigma-Aldrich (St-Louis, MO). For this study, endometrioid carcinoma cases (Type I EmCa, n = 10) and normal proliferative endometrium (n = 10) were selected. Five of the Type I EmCa tissue samples were from biopsies that had been examined in our previous study. For proteomic analysis, tissue samples were obtained from the mirror-face of the residual block used for histopathological evaluation by the pathologist. Tissue samples were washed three times in ∼1 mL of phosphate-buffered saline (PBS) with a mixture of protease inhibitors (1 mM 4-(2-aminoethyl) benzenesulfonyl fluoride, 10 µM leupeptin, 1 µg/mL aprotinin, and 1 µM pepstatin) as described earlier [18]. The washed sample was then homogenized in 0.5 mL of PBS with protease inhibitors using a hand-held homogenizer, flash-frozen in liquid nitrogen, and stored at −80°C until use [18]. After retrieval from storage at −80°C, samples were thawed, clarified by centrifugation, and the protein concentration was determined using Bradford assay (Bio-Rad, CA). For all iTRAQ sets, a reference sample comprising a pool of the ten normal proliferative samples (100 µg lysate from each tissue) was used. For each experiment, 100 µg of proteins were digested with trypsin and labeled individually with the appropriate iTRAQ tag. The assignments of the tags to the sample types were randomized to minimize any potential bias in labeling efficiency between the individual versions of the iTRAQ tags. A total of seven iTRAQ sets were examined, each comprising three of the total of 20 individual samples – ten EmCa and ten normal endometria – and the reference pool. Mouse monoclonal antibodies against calumenin and HNRNPA1 were available from Abcam, cathepsin B from Calbiochem, and S100A6 from Santa Cruz Biotech. Rabbit polyclonal lactate dehydrogenase A (LDHA) was also obtained from Santa Cruz Biotech.

### Strong Cation Exchange (SCX) Separation

Each iTRAQ set was separated by SCX using an HP1050 HPLC instrument (Agilent, Palo Alto, CA) with a 2.1-mm internal diameter x 100-mm-length PolyLC Polysulfoethyl A column packed with 5-µm beads with 300-Å pores (The Nest Group, Southborough, MA) as described previously [18]. Briefly, the iTRAQ set was diluted with the loading buffer (identical in composition to Buffer A: 15 mM KH_2_PO_4_ in 25% acetonitrile, pH 3.0) to a total volume of 1.8 mL, and the pH adjusted to 3.0 with phosphoric acid. The solution was then filtered using a 0.45-µm syringe filter (Millipore, Cambridge, ON, Canada) before loading onto the column. Separation was performed using a linear binary gradient over 1 h, plus 30 min of column re-equilibration (Table 4). As described earlier, Buffer A was identical in composition to the loading buffer; Buffer B was Buffer A containing 350 mM potassium chloride; and Buffer C was Buffer A containing 1 M potassium chloride. Fractions were collected every 2 min using an SF-2120 Super Fraction Collector (Advantec MFS, Dublin, CA) after an initial wait of 2 min to accommodate the void volume. This resulted in a total of 30 SCX fractions per iTRAQ set. These fractions were dried using speed-vac (Thermo Savant SC110 A, Holbrook, NY) and re-dissolved in a minimal volume of 5% acetonitrile in 0.1% formic acid: typically 8 µL for each fraction No. 6–9, 12 µL for No. 10–13, 16 µL for No. 14–16, 20 µL for No. 17–19, 25 µL for No. 20–21, and 30 µL for the last fractions, No. 22–30. Larger volumes in the later fractions were necessitated by the need to completely dissolve the larger salt pellets resulting from their correspondingly higher salt contents. Fractions No. 1–5 were not analyzed as early fractions mostly contained iTRAQ by-products.

**Table 4:** LC gradient for strong cation exchange separations.

Time	Start	2 min.	58 min.	60 min.	65 min.	75 min.	80 min.	90 min.
%B	0%	0%	100%	100%	0%	0%	0%	0%
%C	0%	0%	0%	0%	100%	100%	0%	0%

Buffer A: 15 mM KH_2_PO_4_ in 25% acetonitrile, pH 3.0.

Buffer B: Buffer A containing 350 mM KCl.

Buffer C: Buffer A containing 1 M KCl.

### Reverse-Phase (RP) LC-MS/MS

The SCX fractions No. 6–30 of each iTRAQ set were analyzed by online nano LC-MS/MS using the LC Packings Ultimate instrument (Amsterdam, The Netherlands) fitted with a 10-µL sample loop. The autosampler was used in the microliter pick-up mode. For each sample, 1 µL solution was loaded onto a 5-mm reverse phase (RP) C18 precolumn (LC Packings) at 25 µL/min and washed for 4 min before switching the precolumn in line with the separation column. The separation column used was a 75-µm-internal diameter x 150-mm-length capillary column (Integrafrit capillary from New Objective, Woburn, MA) packed in-house with 3.5-µm C18 beads with 100-Å pores from Kromasil (Akzo Nobel/EKA Chemicals inc, NY). The flow rate used for separation on the RP column was 200 nL/min. Solvent A was 5% acetonitrile in 0.1% formic acid; Solvent B was 95% acetonitrile in 0.1% formic acid. The solvent gradient is detailed in Table 5. A new column was used for each iTRAQ set.

**Table 5:** LC gradient for on-line reverse phase separations.

Time	Start	5 min.	10 min.	120 min.	140 min.	145 min.	155 min.	157 min.	189 min.
%B	5%	5%	10%	35%	60%	80%	80%	5%	5%

Solvent A: 5% acetonitrile in 0.1% formic acid.

Solvent B: 95% acetonitrile in 0.1% formic acid.

Online MS/MS was accomplished on a QSTAR Pulsar hybrid quadrupole/time-of-flight (QqTOF) tandem mass spectrometer (Applied Biosystems/MDS SCIEX, Foster City, CA) in information-dependent acquisition mode with the scan cycles set up to perform a 1-s MS scan followed by five MS/MS scans of the five most-abundant peaks for 2 s each and with a dynamic exclusion period of 30 s. The performance of the LC-MS/MS system was evaluated at minimum once every three days by means of a standard of 80 fmol of bovine serum albumin tryptic digest. Mass calibration of the TOF analyzer was verified at the same time and adjusted when necessary.

### Protein Identification and iTRAQ Ratio Calculation

MS/MS spectra were processed by the software Proteinpilot version 2.0.1 (AB SCIEX, Foster City), using the Paragon algorithm [48] and against a concatenated Swissprot/Panther database of 66082 distinct human protein entries (132164 entries after the reversed sequences were added). Protein identification was performed using a confidence threshold of 95% (Proteinpilot Unused score ≥1.31) with MMTS selected as cysteine modification, and with the search option ‘emphasis on biological modifications’ checked.

Proteinpilot measures the height of the reporter ions' peaks in the MS/MS spectra and calculates the ratios relative to the reporter that is designated as the reference. It then applies a manufacturer-provided value to correct for the isotopic purity of the labels, as well as a data-dependent, automatically generated bias value to correct for discrepancies in sample amounts used for each sample in the set. The individual peptide ratios for each protein are averaged in log space with only high-confidence peptides (confidence ≥95) being used for the calculation of the overall iTRAQ ratio reported. Quantification is performed exclusively through the use of unique peptides, thus eliminating peptides matching more than one protein or common to different isoforms.

In instances where a group of peptides can be assigned to more than one protein, Proteinpilot lists the alternative possibilities under the selected protein identity. In this respect, the peptides are unique to a group of proteins, with the selected identity being the representative protein of this group.

### Iterative Runs

Using the peptide summary report generated by Proteinpilot for a specific set of samples, the *m/z* values and RP elution times of the identified ions (peptides) were imported into the exclusion list of our acquisition method. A tool in Perl language, developed in-house, helped in sorting the peptides and removed most of the redundant entries in the peptide summary report. Tolerance windows were set at ±120 ppm for *m/z* and ±720 s for elution time. The dynamic exclusion list was maintained for a 30-s time window after the first MS/MS scan at any particular *m/z* ratio. Initially, we used the report for the analysis of all 25 fractions to generate a single exclusion list comprising *m/z* values of peptides and their elution times, before starting the second iteration. The report for the second iteration of the same fractions was in turn used to generate a second list that was added to the first exclusion list before the third iteration was performed. After the second iTRAQ set was analyzed, we altered the strategy from using all fractions to generate the exclusion lists to doing so from groups of five fractions. This allowed for a more efficient process with a second group of five fractions being acquired during the computer analysis of the first group. Besides the logistic advantage of this approach, which maximized the use of available mass spectrometer and computer time, the strategy also limited the time interval between the three RP separations of the same fraction. This latter feature reduced the risk of a shift in the elution time due to chromatographic column aging, or worse, replacement following a column failure. A final data analysis was performed on the complete series of the 25 data files to generate a data report encompassing all 25 fractions of each iTRAQ set. The analysis of all 25 fractions as a single group enhanced the number of positive identifications and the identification confidence, by regrouping different peptides of the same protein scattered across many fractions.

### Analysis of differentially expressed proteins in EmCa as biomarkers

Proteins identified in all runs were matched by accession numbers; the expression ratios of proteins in each sample were averaged across different runs and the mean expression ratios of proteins were evaluated, with the help of a script written in Matlab (version 7.7.0.471). Statistical analyses for differential protein expressions in cancerous versus non-cancerous tissue samples were performed on the basis of the following criteria: (1) Proteins had to have iTRAQ ratios determined in at least five of the EmCa samples and five of the normal proliferative samples. (2) The averages of the iTRAQ ratios for the EmCa samples were ≥1.5 or ≤0.67, and the averages for the normal proliferative samples did not exceed the thresholds (i.e., 1.5> normal mean >0.67) as described earlier [18], [19], [49]. In addition, some underexpressed proteins in EmCa were accepted provided they were observed at least three times in the EmCa samples and that all iTRAQ ratios were ≤0.67. This modification was adopted to accommodate the lower chance of determining low-abundance proteins due to the stochastic nature of MS/MS analysis. These proteins, however, still had to be observed five or more times in the normal samples for inclusion. (3) The means of the iTRAQ ratios from the EmCa and normal proliferative samples were compared using the Student *t*-test. Proteins fulfilling all of the above criteria and exhibiting significant differential expressions according to the *t*-test (p≤0.05) were considered to be potential biomarkers for EmCa. The above criteria have proven to work well on a number of cancers: the majority of biomarker candidates identified subsequently verified successfully [18], [19], [49]. To further evaluate the significance of these differentially expressed proteins as biomarkers, PPVs and AUCs were evaluated by means of ROC analyses as described earlier [18], [50].

### Pathway Analysis

Potential novel molecular targets identified in this study (Tables 2 and 3) were subjected to pathway analyses using Ingenuity Pathways Analysis (IPA), version 7.5 (Ingenuity Systems, Redwood City, CA). This software interrogated a proprietary database of published data to generate protein interaction networks. The IPA database consists of exclusive ontology representing 300 000 biologic objects ranging from genes, proteins, and molecular and cellular processes. More than 11,200 human genes are currently represented in the database. The proteins were categorized based on location, cellular components, and reported or suggested biochemical, biologic and molecular functions using the software. The identified proteins were then mapped to networks that were generated based on evidence from existing literature available in the Ingenuity database and then ranked by score. A score of 3 or higher has a 99.9% confidence level of not being generated by random chance alone and was used as the cutoff for identifying protein networks.

### Western blotting of differentially expressed proteins in EmCa tissues

Whole-cell lysates from normal endometrium and EmCa tissues used for iTRAQ analysis was used for verification of cathepsin B, calumenin, S100A6, lactate dehydrogenase A (LDHA), and HNRNPA1 proteins. Equal amounts of proteins (50 µg/lane) were resolved on 10% sodium dodecyl sulfate (SDS) polyacrylamide gel. The proteins were then electro-transferred onto polyvinylidene difluoride (PVDF) membrane (BioRad, Hercules, CA). After blocking with 5% non-fat powdered milk in Tris-buffered saline (TBS, 0.1 M, pH = 7.4), blots were incubated with respective mouse monoclonal antibody at appropriate dilutions at 4°C overnight. The abundance of β-actin (mouse monoclonal antibody, Cell Signaling Tech.) served as a control for protein loading in each lane. Membranes were incubated with secondary antibody, horseradish peroxidase-conjugated mouse/rabbit anti-IgG (BioRad, CA), diluted at an appropriate dilution in 1% bovine serum albumin, for 2 h at room temperature. After each step, blots were washed three times with Tween (0.1%)-TBS. Protein bands were detected by the enhanced chemiluminescence method (GE Health Care) on Amersham hyperfilm [50].

### Immunohistochemical analysis of S100A6 in normal endometrium and EmCa tissues

Paraffin-embedded sections (4 µm) of human normal endometrial tissues and Type I endometrial cancer (n = 5 each) were collected on snow-coat slides. In brief, the sections were deparaffinized in xylene, hydrated in gradient alcohol, and pre-treated in a microwave oven for 15 min at maximum power in citrate buffer (0.01 M, pH = 6.0, 0.5% Tween-20) for antigen retrieval. The sections were incubated with hydrogen peroxide (0.3% v/v) in phosphate buffer saline (PBS) for 15 min to quench the endogenous peroxidase activity, followed by blocking with 5% fetal bovine serum to preclude non-specific binding. Thereafter, the slides were incubated with mouse monoclonal anti-S100A6 antibody (sc-52948, 1∶50 dilution, Santa Cruz Biotechnology, CA) for 16 h at 4°C. The primary antibody was detected using the streptavidin-biotin complex with the Dako LSAB plus kit (Dako Cytomation, Glostrup, Denmark) and diaminobenzidine as the chromogen as described earlier by us [50]. All procedures were carried out at room temperature unless otherwise specified. Slides were washed three times using TBS with 0.025% Triton-X after every step. Finally, the sections were counterstained with Mayer's hematoxylin and mounted with DPX mountant. In the negative control tissue sections, the primary antibody was replaced by isotype specific non-immune mouse IgG. The sections were evaluated by light microscopic examination.

## Supporting Information

Figure S1 **MS/MS spectrum of peptide LQDAEIAR from protein S100-A6.**
(TIF)

Figure S2 **MS/MS spectrum of peptide VCPFAGILENGAVR from beta-2 glycoprotein 1.**
(TIF)

Table S1 **Protein average iTRAQ ratios and t-test results.**
(XLS)

Table S2 **Individual iTRAQ ratios of biomarker candidates.** Average ratios per sample: C =  endometrial carcinoma, N =  normal proliferative endometrium. The proteins from the tables S2a and S2b here were reported in Tables 2 and 3 in the article, respectively.
(XLS)

Table S3 **Confidence of identified peptides.** A - Identified peptides whose Proteinpilot confidence was greater than 95%. Proteins identified by a single peptide were sorted on top of the table. #Occurences  =  number of times a particular peptide has been identified over all sample sets. At the bottom of this table, summary of the number of peptides per proteins. B - Identified peptides whose Proteinpilot confidence was greater than 99%.
(XLS)

Table S4 **Samples in which each protein from** **Table 2** **has been identified.**
(XLS)
