# Supplementary figures and images for: Identification of Novel Molecular Targets for Endometrial Cancer Using a Drill-Down LC-MS/MS Approach with iTRAQ
Source: PLoS One. 2011 Jan 31;6(1):e16352. doi: 10.1371/journal.pone.0016352 (PMC3031560; doi:10.1371/journal.pone.0016352)

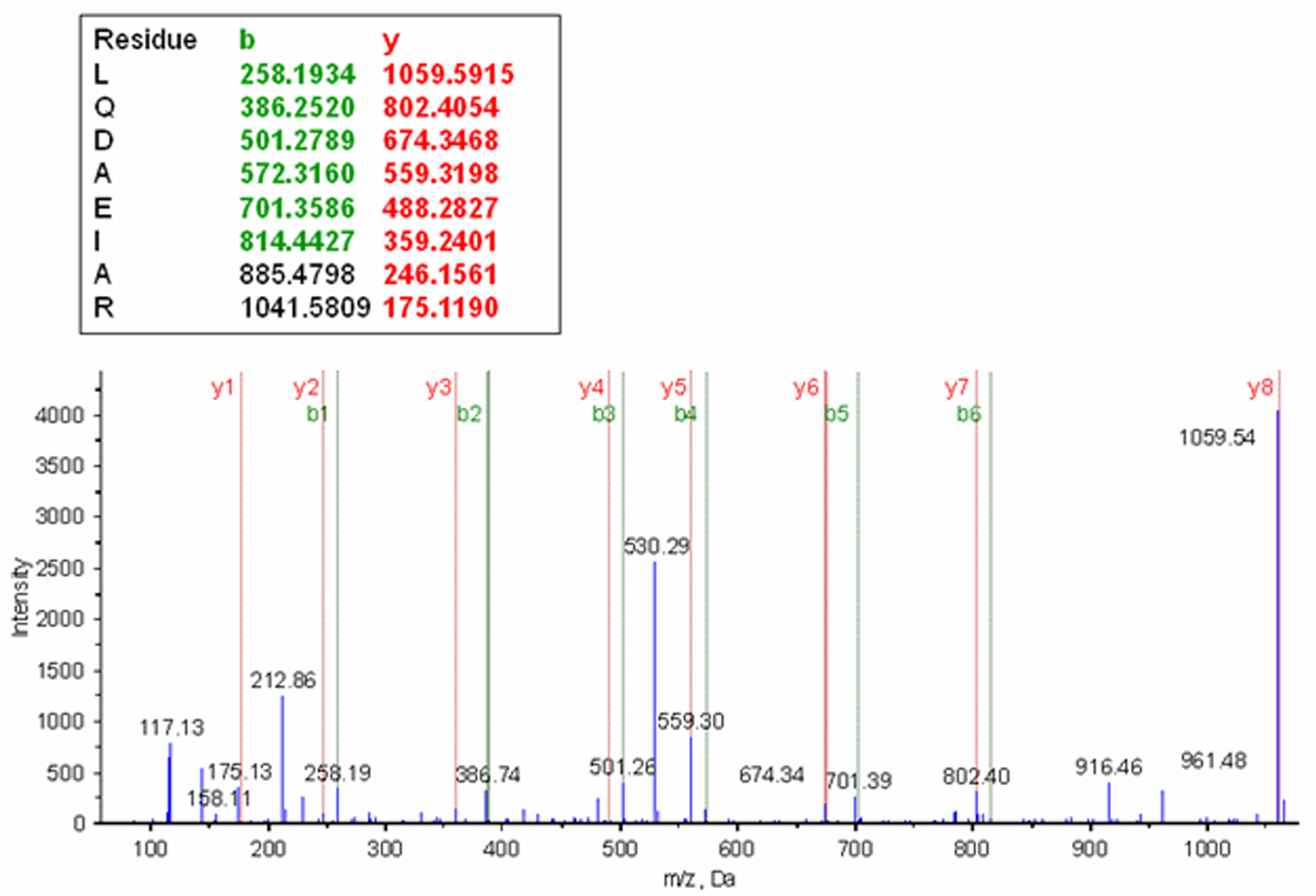

Supplement: Figure S1 — MS/MS spectrum of peptide LQDAEIAR from protein S100-A6. (TIF) [file pone.0016352.s001.tif]

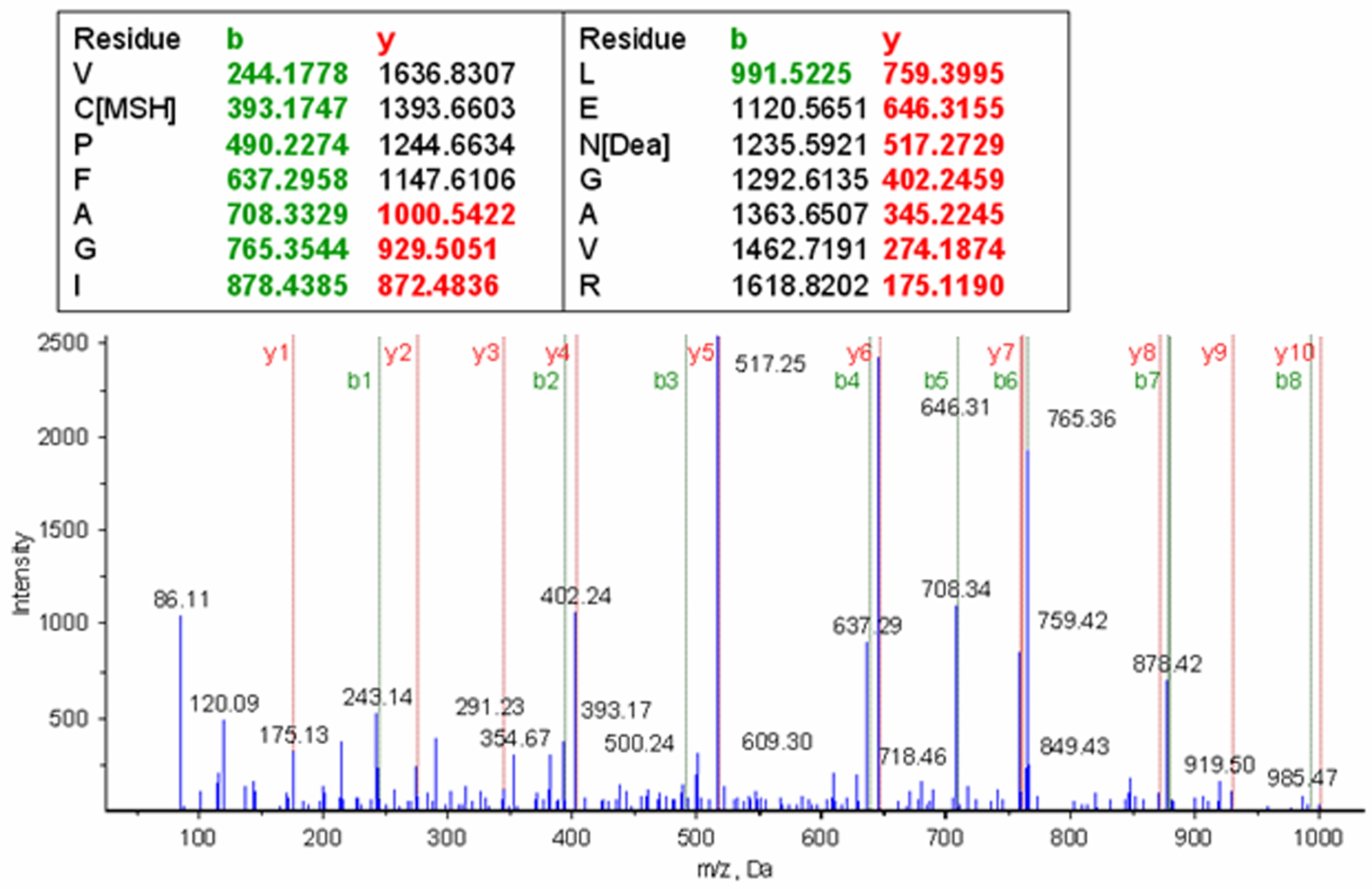

Supplement: Figure S2 — MS/MS spectrum of peptide VCPFAGILENGAVR from beta-2 glycoprotein 1. (TIF) [file pone.0016352.s002.tif]
